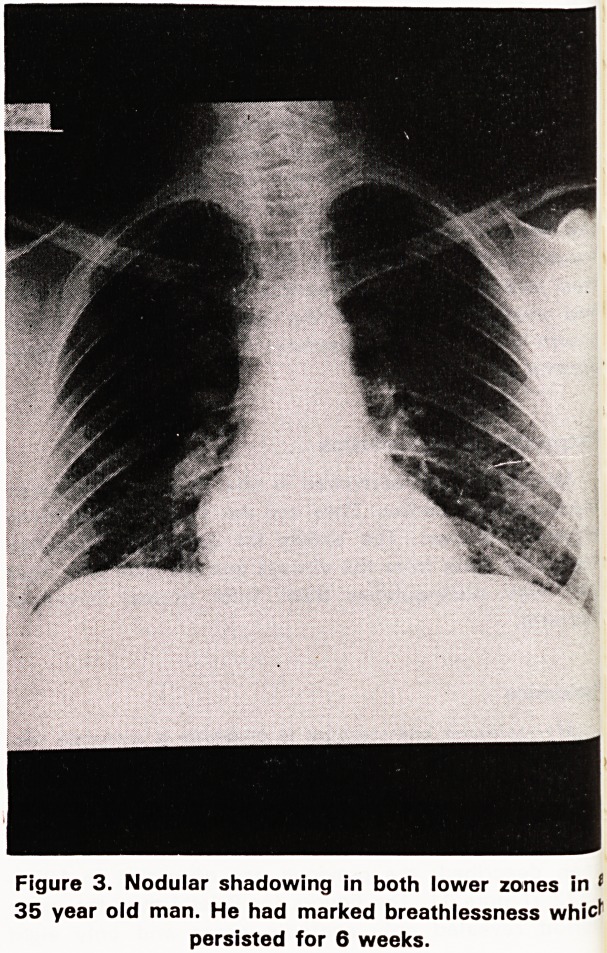# Mycoplasma Pneumonia in Adults

**Published:** 1977

**Authors:** Roger White

**Affiliations:** Frenchay Hospital


### Mycoplasma Pneumonia in Adults

**Roger White, MA, MD, MRCP**
Frenchay Hospital

### Introduction

The organism *Mycoplasma pneumoniae* is responsible for both upper and lower respiratory infections in man. In 1974 and 1975 there was an epidemic of mycoplasma infection in the British Isles and many cases were indentified in the Bristol area. It is certain that many were not diagnosed and that appropriate treatment was not given. It therefore seems opportune to review the features of this infection with particular reference to pneumonia where there are likely to be therepeutic implications. One of the important aspects of the infection is that it appears to respond only to tetracyclines and to erythromycin.

### History

During the 1930's a type of pneumonia which differed from the classical pneumococcal pneumonia was being reported. No specific cause for this pneumonia could be identified but over one half of the patients developed cold agglutinins in the blood. The title of Primary Atypical Pneumonia was given to this disease. In 1944 an agent was isolated by Eaton which was found to transmit the disease from man to animals. It was able to pass through bacterial filters and was therefore thought to be a virus. It became known as the Eaton Agent. Subsequently the organism was shown to be susceptible to certain antibiotics and eventually in 1962 the agent was isolated and grown on a cell-free medium. It was renamed *Mycoplasma pneumoniae.*

### Properties of Mycoplasma

*Mycoplasma pneumoniae* is one of the eight human mycoplasma species. They are the smallest free-living organism known. The ability for cell-free replication separates them from the viruses and the lack of a rigid cell wall distinguishes them from normal forms of bacteria.

### Incidence

*Mycoplasma pneumoniae* is probably a common respiratory pathogen. A study of normal people in Southern England showed serological evidence of past infection in 19 per cent (Lambert, 1968). It is endemic in the community but several epidemics have been described, the previous one occurring in 1971-1972 (Figure 1). The figures for Bristol over the same period revealed only sporadic cases and only eight

**Figure 1. Case reports of mycoplasma infection in Great Britain 1969-1976 (Communicable diseases report 1976)**

patients were identified in 1971-1972. It seems likely that cases have not been identified rather than that the disease has not been occurring.

In the twelve months between August 1974 and July 1975 there were ninety-six definite instances of infection due to *Mycoplasma pneumoniae* in the Bristol area. Forty-seven patients were children and forty-nine were adults. Almost all of of the adults had pneumonia. In the Frenchay district *Mycoplasma pneumoniae* was responsible for 40 per cent of primary pneumonia in adults seen during that year. Subsequently there have been sporadic cases, mainly in young adults.

### Clinical Features

The majority of illnesses caused by this organism are mild upper respiratory infections and acute bronchitis. It is probable that many people infected remain asymptomatic. Many patients will never seek medical attention at all and most who do will be dealt with by their general practitioner. A proportion of those infected, perhaps one in twenty of adolescents and young adults, but possibly a higher number of children, develop pneumonia (Jones, 1971). From this it will be appreciated that the patients seen in hospital represent only a small proportion of those with mycoplasma infection within the community and are likely to have been selected by the severity or prolonged nature of their symptoms. The typical history is of an influenza-like illness with headaches, sore throat, muscle aches and fever. The headache can at times be severe and may initially suggest meningitis. In the beginning there is a dry cough but sputum production usually starts during the next day or two. In most cases the sputum is mucoid and this is an important sign that the penumonia is unlikely to be bacterial in origin. It often becomes mucopurulent after a few days. Sometimes the sputum is mucopurulent from the onset and distinction from bacterial pneumonia becomes more difficult. Pleuritic chest pain has been recorded by others (Putman, et al, 1975) but I have never seen this occur. The presence of pleurisy should be regarded as being more indicative of a bacterial infection. The duration of the illness is very variable, but the majority recover within two weeks. Most of the patients diagnosed during the recent epidemic have had more prolonged or unusually severe illnesses and this is the reason that they have been referred to hospital. Frequently there has been a history of persistent fever with associated cough and sputum. The symptoms have not responded to ampicillin or to trimethoprim/sulphonamide and there are persistent signs of consolidation in the lung. The introduction of tetracycline even at this late stage has regularly resulted in a fall in the temperature within twenty-four hours and usually there is rapid improvement of other symptoms. In some patients the cough is slow to clear in spite of appropriate drug treatment, and the pulmonary consolidation has at times taken up to three months to resolve. Similarly to many virus infections the illness may be followed by several weeks of general debility.

**Radiographic Appearances**

There are no appearances which are typical. The usual findings are of a lobar or segmental consolidation. Any lobe can be affected and other lobes on the same or opposite side are often involved at the same time. At times the appearance may mimic tuberculosis (Figure 2). A few patients develop a different pattern. They have nodular consolidation affecting both lower zones which does not become confluent (Figure 3). This pattern of consolidation is unlike that of a bacterial pneumonia and the reason for this may be that the bronchioles and smaller airways are affected. Lung function tests may demonstrate airways obstruction, suggesting that some of the inflammation is proximal to the alveoli. These patients have a more severe degree of illness and breathlessness is prominent. With this variety of infection the symptoms are also slow to clear.

**Figure 2. Right upper lobe shadowing mimicking tuberculosis in a 14 year old boy.**

**Figure 3. Nodular shadowing in both lower zones in a 35 year old man. He had marked breathlessness which persisted for 6 weeks.**

### Diagnosis

The diagnosis is made by the demonstration of complement fixing antibodies to *Mycoplasma pneumoniae* in the serum. A fourfold rise in the titre of antibodies is traditionally regarded as diagnostic but a titre of 256 or more can be regarded as confirmation. A single high titre is the most that can be expected if blood is first taken after three weeks of illness. Antibodies usually start to develop during the second week of the illness but their appearance may be delayed and one of the Bristol patients did not develop a high titre until the fourth week. Cold agglutinins are present in the blood in about 40 per cent of patients. These are always suggestive of mycoplasma infection but are not specific. The titre may be small and reported as not significant by the laboratory but the presence of cold agglutinins in any titre is suggestive of mycoplasma infection if the symptoms are compatible.

*Mycoplasma pneumoniae* can be cultured from throat swabs but the technical difficulties make this method unreliable unless its is regularly done by the laboratory. It is slow-growing, taking up to three weeks, by which time the diagnosis will usually have become apparent from the serological tests. Culture of the organism is not suitable therefore as a diagnostic test, but will provide retrospective confirmation of single high titres.

### Other Laboratory Investigations

The white blood cell count is variable. The total count is rarely more than 16.0 x $10^9$/l but a moderate neutrophilia is common. A lymphocytosis or leucopenia is not characteristic of mycoplasma infection. Two patients in the recent epidemic had a moderate eosinophilia. The plasma viscosity is often more than 2.0 at the beginning of the illness.

### Treatment

The organism is sensitive to antibiotics of the tetracycline group and to erythromycin. One trial of therapy has suggested that demethylchlortetracycline is the best antibiotic (Shames, et al, 1970) but in practice oxytetracycline appears to be effective. In children tetracyclines should, of course, be avoided but erythromycin can be used instead. Other antibiotics which are commonly used in respiratory infections such as the penicillins, trimethoprim/sulphonamide and cephalosporins are ineffective. Many patients will not need antibiotics because of the mild nature of the illness. In others the diagnosis will only be made when the symptoms have cleared and no treatment is required in these patients.

### Complications

One case of possible lung abscess has been recorded (Siegler, 1973). The commonest complications are outside the respiratory tract. Haemolysis occurs in about 5 per cent of patients (Jones, 1971). This is due to the presence of cold agglutinins but its occurrence appears to be unrelated to the titre. It begins in the second or third week and may not occur until after the respiratory symptoms have subsided. This complication should be borne in mind in any patient who presents with symptoms of anaemia and who gives a history of recent respiratory infection. Erythema multiforme is the most frequent skin disease associated and has been recorded as being due to mycoplasma in the absence of respiratory symptoms (Gordon and Leyall, 1970).

An acute arthritis can occur. A number of joints are usually involved and it may be mistaken for rheumatoid arthritis or rheumatic fever. I have not seen this in association with pneumonia and it is more likely to present as a separate entity.

### Discussion

It can be assumed that the great majority of instances of mycoplasma infection are not diagnosed and that the illness is of a mild nature, not requiring antibiotics. The symptoms of mycloplasma pneumonia are not specific to that condition and are similar to those occurring in various viral pneumoniae.

Antibiotics are frequently prescribed in patients suffering from viral pneumonia because of the difficulty in distinguishing them with certainty from the bacterial pneumonias. In adult pneumonia where the symptoms and signs are not suggestive of an acute pneumococcal lobar pneumonia there is a good case to be made for the routine use of the tetracyclines. As well as being effective in mycoplasma infection they will also suppress the occasional pneumonia due to Q fever and psitticosis. In most instances a tetracycline will be sufficient for the bacterial pneumoniae which may complicate virus infections such as influenza.

### Acknowledgement

All of the virology investigations have been done at the Public Health Laboratory, Bristol, and I am grateful to Dr. Suzanne Clarke for her valuable help.

### References

Communicable Diseases Report (1976). Public Health Laboratory Service, No. 17.

Gordon, A. M. and Lyell, A. (1970). Mycoplasmas and their association with skin disease. British Journal of Dermatology. **82, 414.**

Jones, M. C. (1971). Mycoplasma infections of the respiratory tract. British Thoracic and Tuberculosis Association Review. **1**, 1.

Lambert, H. P. (1968). Mycoplasma pneumonia infections. In Symposium on Acute Respiratory Diseases. Journal of Clinical Pathology. **21**, Supplement 2, 52.

Putman, C. E., Curtis, A. McB., Simeone, J. F. and Jensen, P. (1975). Mycoplasma pneumonia. American Journal of Roentgenology. **124, 417.**

Shames, J. M., George, R. B., Holliday, W. B., Rasch, J. R. and Mogabgab, W. J. (1970). Comparison of antibiotics in the treatment of mycoplasma pneumonia. Archives of Internal Medicine. **125, 680.**

Siegler, D. I. (1973). Lung abscess associated with Mycoplasma pnemoniae infection. British Journal of Diseases of the Chest. **67, 123.**

## Figures and Tables

**Figure 1. f1:**
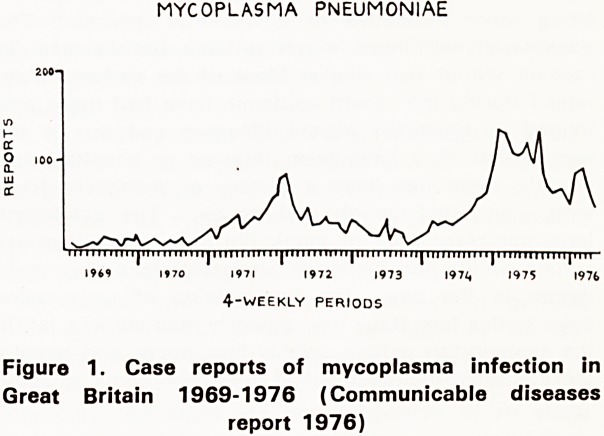


**Figure 2. f2:**
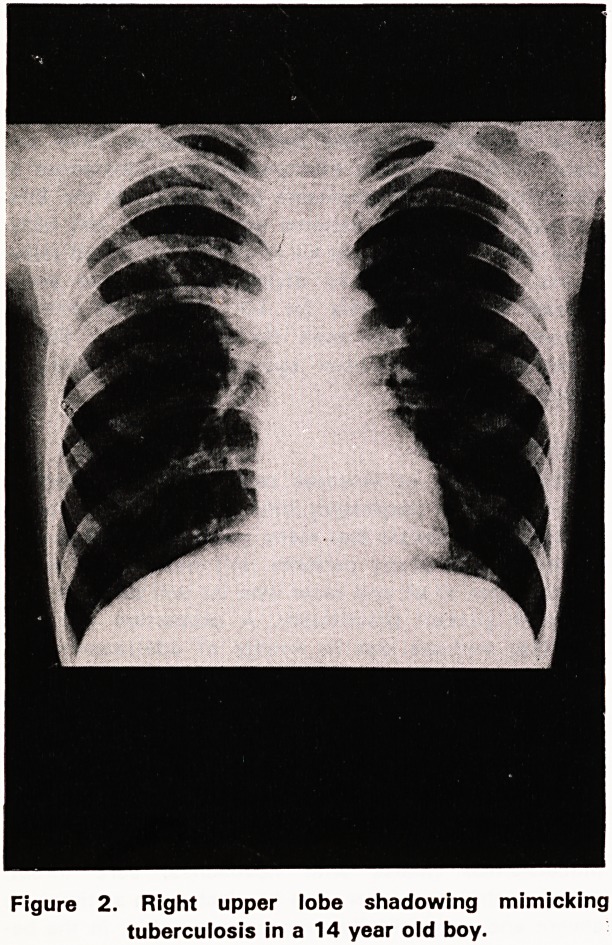


**Figure 3. f3:**